# Neuroprotective Effects of Selected Microbial-Derived Phenolic Metabolites and Aroma Compounds from Wine in Human SH-SY5Y Neuroblastoma Cells and Their Putative Mechanisms of Action

**DOI:** 10.3389/fnut.2017.00003

**Published:** 2017-03-14

**Authors:** A. Esteban-Fernández, C. Rendeiro, J. P. E. Spencer, D. Gigorro del Coso, M. D. González de Llano, B. Bartolomé, M. V. Moreno-Arribas

**Affiliations:** ^1^Instituto de Investigación en Ciencias de la Alimentación (CIAL), CSIC-UAM, Madrid, Spain; ^2^Department of Food and Nutritional Sciences, School of Chemistry, Food and Pharmacy, University of Reading, Reading, UK; ^3^Beckman Institute for Advanced Science and Technology, University of Illinois Urbana–Champaign, Champaign, IL, USA

**Keywords:** wine, polyphenols, gut phenolic metabolites, aroma compounds, mitogen-activated protein kinase, neuroprotection

## Abstract

Moderate wine consumption has shown the potential to delay the onset of neurodegenerative diseases. This study investigates the molecular mechanisms underlying the protective effects of wine-derived phenolic and aroma compounds in a neuroinflammation model based on SIN-1 stress-induced injury in SH-SY5Y neuroblastoma cells. Cell pretreatment with microbial metabolites found in blood after wine consumption, 3,4-dihydroxyphenylacetic (3,4-DHPA), 3-hydroxyphenylacetic acids and salicylic β-d-O-glucuronide, at physiologically concentrations (0.1–10 μM) resulted in increased cell viability versus SIN-1 control group (*p* < 0.05). Results also showed significant decreases in mitogen-activated protein kinase (MAPK) p38 and ERK1/2 activation as well as in downstream pro-apoptotic caspase-3 activity by some of the studied compounds. Moreover, pretreatment with p38, MEK, and ERK1/2-specific inhibitors, which have a phenolic-like structure, also resulted in an increase on cell survival and a reduction on caspase-3 activity levels. Overall, these results contribute with new evidences related to the neuroprotective actions of wine, pointing out that wine-derived human metabolites and aroma compounds may be effective at protecting neuroblastoma cells from nitrosative stress injury by inhibiting neuronal MAPK p38 and ERK1/2, as well as downstream caspase 3 activity.

## Introduction

Oxidative and nitrosative stress play important roles in the development of neurodegenerative diseases such as Alzheimer’s and Parkinson’s (1). While the harmful effects of heavy alcohol intake are well-established (2), several epidemiological studies have suggested that low to moderate consumption of red wine (~250 mL per day) can be beneficial in delaying the onset of cognitive impairments in aging and neurodegenerative diseases (3–8). In agreement with this, evidence from human randomized controlled trials shows that acute supplementations with specific wine compounds, such as anthocyanins or flavonoids, report improvements in recognition and working memory, attention, and psychomotor function (5, 9). Moreover, *in vivo* studies in rodent models further support the idea that moderate red wine intake can impact cognitive function (10–12). In particular, replacement of water with a red wine (20% ethanol v/v) enriched in flavan-3-ols (0.003–1.53 g/L) and anthocyanins (0.648 g/L), resulted in an improved cognitive performance when compared to 20% ethanol control group in rodents (10). Similarly, the intake of red wine during 7 months showed to be protective in an Alzheimer’s mice model, resulting in a reduction of cognitive deterioration and neuropathology, as measured by spatial memory tests and Aβ peptides clearance in Tg2576 mice (11, 12).

Red wine is a complex matrix rich in polyphenols, in particular flavan-3-ols, and also an important source of aroma compounds. Flavan-3-ols are metabolized in the human colon by microbial catabolism reactions (i.e., hydrolysis, oxidation), originating metabolites such as propionic acid, phenylacetic acid, and benzoic acids derivatives (13). Further metabolism in the liver results in an extensive conjugation into glucuronides, sulfates, and *O*-methyl derivatives. More importantly, these metabolites have been detected in human plasma at physiological micromolar concentrations following red wine intake (14). Several studies have further demonstrated an ability of gut-derived metabolites to cross the blood–brain barrier (BBB) both in *in vitro* and *in vivo* models (15–17), suggesting that these might be responsible of the described protective effects of red wine polyphenols in brain function and cognitive performance. On the other hand, wine aroma compounds, such as linalool and 1,8-cineole, have been extensively described for their antioxidant, anti-inflammatory, and antimicrobial properties in *in vitro* models (18, 19). Interestingly, wine-derived aroma compounds are small lypophilic molecules, which have also been shown to cross the BBB and might equally contribute to beneficial effects in the central nervous system (20), although the underlying mechanism of action has not been fully characterized (21, 22).

Despite the increasing evidence of the potential of red-wine polyphenols to affect brain function, the underlying mechanisms by which these compounds might influence neuronal function remain to be established (23). Both, *in vitro* and *in vivo* studies suggest an ability of polyphenols, such as flavonoids, to interact with signaling pathways that modulate neuronal stress-induced apoptosis (24), including the nuclear factor-ĸB or mitogen-activated protein kinase (MAPK) pathways (in particular ERK1/2, JNK, and p38) (23, 24). These are known to activate downstream signals, such as STAT-1 (activator of transcription-1), related to pro-inflammatory responses in neurons (25, 26) as well as caspase-3 proteases activity, pro-apoptotic marker of cell death (27). While the interaction of flavonoids with such signaling pathways has been previously studied (1, 28), the effect of the physiological relevant human gut-derived metabolites (particularly phenolic acids) in neuronal function has been poorly explored.

3-Morpholinosydnonimine (SIN-1), a peroxynitrite generator described as inducing phosphorylation of protein tyrosine residues in brain cells (29), has been used as a neuronal damage inductor. It is able to produce peroxynitrite (ONOO^−^) from nitrogen monoxide (NO) and superoxide anion $\left({\text{O}}_{2}^{\cdot -}\right)$. ONOO^−^ is one of the most reactive species implicated in causing damage in several cellular functions (i.e., oxidation of DNA, lipids, and protein sulfhydryls or the nitration of DNA and tyrosine) (30). In the present study, SIN-1-induced nitrosative stress in a human neuroblastoma cell line (SH-SY5Y) was used as a model of neuroinflammation (29, 30) in order to study the protective effects of wine-derived human gut metabolites, mainly phenolic compounds (3,4-dihydroxyphenylacetic, 3-(4-hydroxyphenyl)propionic, 3-hydroxyphenylacetic (3HPA), and 3-(3-hydroxyphenyl) propionic acid, salicylic acid, β-d-*O*-glucuronide of salicylic acid) and aroma compounds (linalool and 1,8-cineole) on neuronal survival, as well as their ability to interact with MAPK pathways (ERK1/2, JNK, p38) and downstream signaling processes (STAT 1, caspase-3).

## Experimental

### Chemicals

3,4-Dihydroxyphenylacetic acid (3,4-DHPA) and 3-(3-hydroxyphenyl) propionic acid (3HPP) were purchased from Alfa Aesar (Ward Hill, MA, USA). 3-(4-hydroxyphenyl) propionic acid (4HPP) was obtained from Sigma-Aldrich (Saint Louis, MO, USA), and 3HPA was purchased from Merck (Darmstadt, Germany). Salicylic acid (2-hydroxybenzoic acid) and its β-d-O-glucuronide were from NE Scientific (Natick, MA, USA). Linalool and 1,8-cineole were purchased in Sigma-Aldrich (Saint Louis, MO, USA). Stock solutions of these compounds were freshly prepared and filtered (0.22 μM) in sterile PBS (1,000 μM). 3-Morpholinosydnonimine (SIN-1) was acquired from Alexis Biochemicals, Enzo Life Sciences (Exeter, UK). Anti-GADPH was obtained from New England Biolabs (Hitchin, UK). Primary rabbit polyclonal antibodies specific for MAPK signaling including total SAPK/JNK and phospho-SAPK/JNK (Thr^183^/Tyr^185^), total p38 and phospho-p38 (Thr^180^/Tyr^182^), total ERK1/2 and phospho-ERK1/2 (Thr^202^/Tyr^204^), and for total STAT-1 and phospho-STAT-1 (Tyr^701^) were from New England Biolabs (Hitchin, UK). Horseradish peroxidase (HRP)-conjugated goat anti-rabbit secondary antibody was obtained from Sigma-Aldrich (Saint Louis, MO, USA). ECL^®^ reagent and Hybond nitrocellulose membrane were purchased from Amersham Biosciences (Amersham, UK). All tissue culture reagents were purchased from Lonza (Slough, UK) and Invitrogen (Paisley, UK). MTT reagent was obtained from Sigma-Aldrich (Saint Louis, MO, USA).

### Cell Culture

SH-SY5Y (ATCC^®^ CRL2266™) human neuroblastoma cell line was purchased from the American Type Culture Collection. Cells were routinely grown in 75 cm^2^ flasks in a mixture of Dulbecco’s modified Eagle Medium and Ham’s F12 (1:1 v/v) (Lonza, Slough, UK) supplemented with 10% fetal bovine serum, antibiotics (100 IU/mL penicillin and 100 μg/mL streptomycin) and 1% non-essential aminoacids (37°C and 5% CO_2_). Twenty-four hours prior to the experiments, cells were detached from flasks with trypsin-EDTA solution (Sigma-Aldrich, Saint Louis, MO, USA) and seeded on the respective plates.

### Assessment of Cell Viability

Neuronal cells (4.10^5^ cells/mL) were seeded on 96-well plates 24 h prior to the incubation with the phenolic acids and aroma compounds (0.1–10 μM). After 18 h, cells were washed twice with DPBS solution (Lonza, Slough, UK) and Thiazolyl Blue Tetrazolium Bromide reagent (MTT) (0.5 mg/mL, final concentration) (Sigma-Aldrich, Saint Louis, MO, USA) was added. Then, plates were returned to the incubator (37°C, 5% CO_2_) for 3 h. Supernatant was carefully removed and formazan crystals were dissolved with pure DMSO before absorbance was measured (570 nm) on a Multiskan multiplate reader (Thermo Fisher, Waltham, MA, USA). Control (no compound added) was considered as maximum of percentage of viability (100%), and the sample values were calculated as: % viability = (Abs_sample_/Abs_control_) × 100. Assays were performed in triplicate.

### Neuroprotective Effect against SIN-1-Induced Cell Death

3-morpholinosydnonimine (SIN-1), a peroxynitrite generator has been used as a neuronal damage inductor. For the assays, neuronal cells (4 × 10^5^ cells/mL) were seeded on 96-well plates 24 h prior to the incubation with the phenolic acids and aroma compounds (0.1–10 μM; 18 h). Then, neuroblastoma cells were exposed to freshly prepared 1 mM SIN-1 for 0–15 h (37°C, 5% CO_2_) and MTT reagent was added (0.5 mg/mL). Plates were returned to the incubator for 3 h, and assay was performed as described above.

For MEK (PD98059, Calbiochem), p38 (SB203580, Calbiochem), and ERK (FR180204, Sigma-Aldrich) inhibitors, the same procedure was applied with the exception that neuroblastoma cells were pretreated for 1 h with different concentrations of inhibitor (0.5–50 μM) prior the exposition to freshly prepared SIN-1 (1 mM) during 0–15 h. Assays were carried out in triplicates.

### Modulation of MAPK Signaling Pathway

To determine the ability of the phenolic acids and aroma compounds to modulate SIN-1-induced MAPK and STAT-1 phosphorylation, neuronal cells (10^6^ cells/mL) were seeded on 6-well plates 24 h prior to incubation with the compounds (0.1–10 μM; 18 h). After this time, cells were activated with SIN-1 (500 μM) for 1–2 h. Then, neuronal cells were first washed with ice-cold PBS with 200 μM EGTA and 200 μM EDTA and lysed on ice using 50 mM Tris, 0.1% Triton X-100, 150 mM NaCl, and 2 mM EGTA/EDTA, containing Complete^®^ Protease Inhibitor Cocktail tablets and PhosStop^®^ Phosphatase Inhibitor Cocktail Tablets (Roche, Basel, Switzerland). Lysed cells were scraped, left on ice to solubilize for 45 min, and total protein concentration was determined by BCA assay (Pierce Kit, Thermo Scientific, Rockford, IL, USA) to normalize protein level. Samples were frozen at −80°C until further immunoblotting analysis.

### Western Immunoblotting

Immunoblotting was performed as previously described (25). Samples (20 μg protein/lane) were run on 10% pre-cast SDS-polyacrylamide gels (Bio-Rad, Hemel Hempstead, UK), and proteins were transferred to nitrocellulose membranes (Hybond-ECL) (Amersham, Buckinghamshire, UK) by semi-dry electroblotting (0.8 mA/cm^2^). Blots were incubated overnight at 4°C, with the primary antibodies described above (all 1:1,000 dilution) in Tris–Tween Buffered Saline (TTBS) containing 1% (w/v) skimmed milk powder antibody buffer, on a three-dimensional rocking table. Afterward, the blots were washed with TTBS, incubated with goat anti-rabbit IgG conjugated to HRP secondary antibody (1:1,000 dilution) for 45 min, washed with TTBS and then exposed to ECL-reagent^®^ for 1.5 min and developed using ImageQuant™ LAS mini 4000 (GE Healthcare, Buckinghamshire, UK). Bands were analyzed using ImageQuant™ Software (GE Healthcare, Buckinghamshire, UK). Molecular weights of the bands were calculated from comparison with prestained molecular weight markers (M_W_ 10–250 kDa) (BioRad, Hemel Hempstead, UK), which were run in parallel with the samples. The equal loading and efficient transfer of proteins was confirmed by using GADPH as internal control.

### Caspase-3 Activity

Neuroblastoma cells (10^6^ cells/mL) were seeded on 6-wells plates 24 h prior the pretreatment with wine constituents (0.1–10 μM; 18 h) or MAPK inhibitors (0.5–50 μM; 1 h) exposure to SIN-1 (1 mM; 6 h). Culture was then washed twice with ice-cold PBS + EGTA + EDTA (200 μM), and the cells were lysed and collected as previously described. The activity of caspase-3-like proteases in the lysates was determined using the caspase-3 colorimetric assay kit (Sigma, Poole, Dorset, UK) according to the manufacturer’s protocol, with the exception that 30 μL of cell lysate was used in assays. Absorbance data (405 nm) obtained using the caspase-3 inhibitors were subtracted from the absorbance obtained without caspase-3 inhibitor to correct for any non-specific hydrolysis. Vehicle controls and blanks were incorporated, and caspase-3 protein was used as a positive control of the assay. Assays were carried out in triplicates.

### Statistical Analysis

Data were expressed as the mean ± SEM of three independent experiments. Statistical analysis was made using one-way analysis of variance, followed by a *post hoc* Dunnett test of each time point to look for significant differences with respect to SIN-1 condition (positive control). Significance level was set at *p* < 0.05. Statistics analysis was carried out using STATISTICA program for Windows, version 7.1 (StatSoft. Inc., 1984–2006).

## Results

### Ability of Phenolic Acids and Aroma Compounds to Protect Neuroblastoma Cells against SIN-1-Induced Neurotoxicity

**Figure 1:**
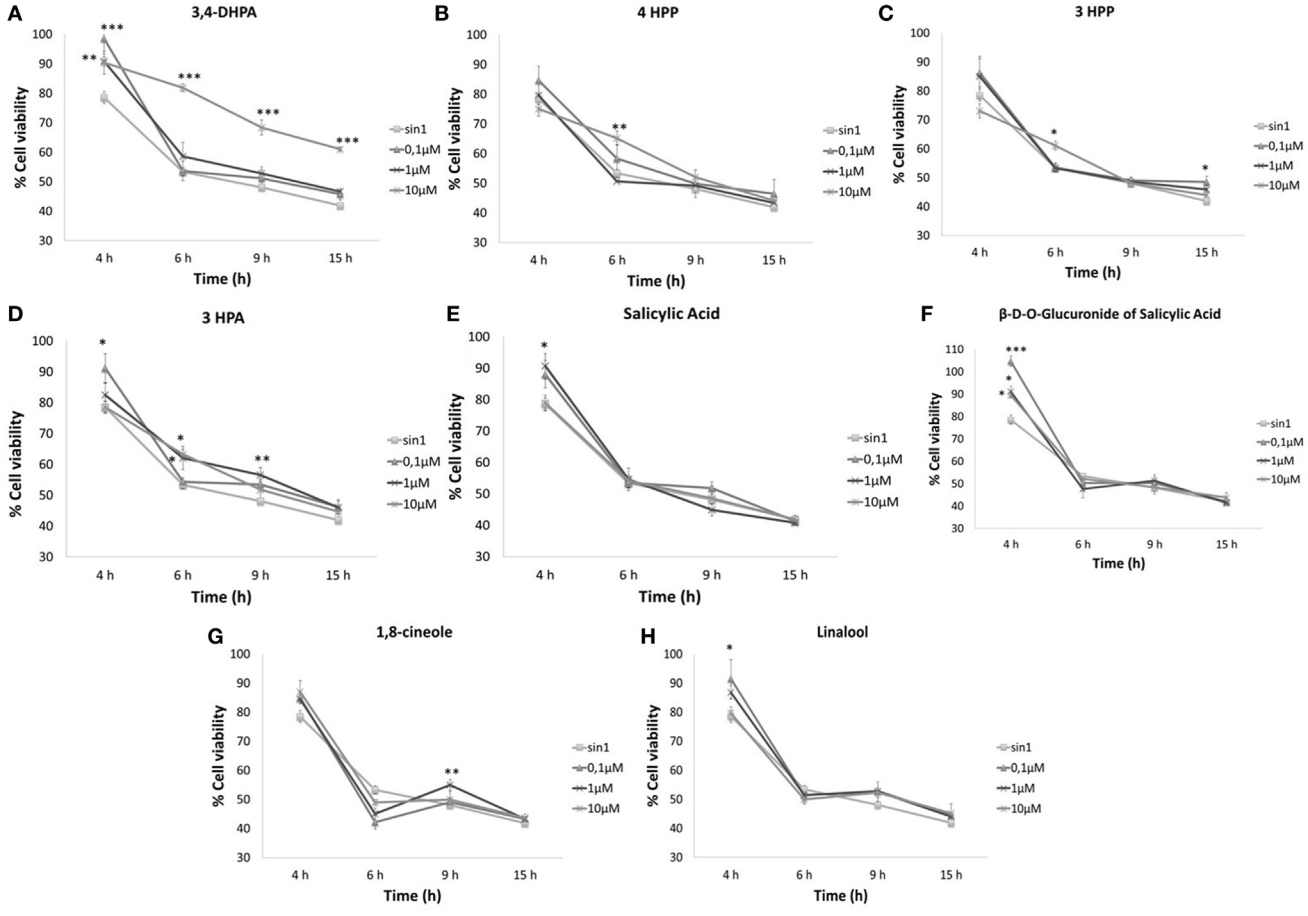
**Neuroprotective effects of phenolic acids (A–F) and aroma compounds (G,H) against SIN-1-induced damage in SH-SY5Y cells**. Following pretreatment with wine compounds (0.1–10 μM) or media (control) for 18 h, cells were exposed to SIN-1 (1 mM) for 0–15 h, before assessment of viability (%) by MTT assay. Results are expressed as means of three independent experiments ± SEM. *p < 0.05, **p < 0.01, ***p < 0.001 indicate values significantly different from SIN-1 group as analyzed by one-way ANOVA followed by Dunnett comparison test.

None of the selected compounds (0.1–10 μM) affected cell viability (%) (data not shown) in relation to the control (no added compound). Pretreatment with 10 μM 3,4-DHPA acid resulted in a significant increase in cell viability against SIN-1-induced neuronal death (Figure 1A) when compared to the SIN-1 control group, regardless of the length of time of exposure to SIN-1 (4 h: *p* < 0.01; 6–15 h: *p* < 0.001). The lower concentrations of 3,4-DHPA (<10 μM) were also effective, but only during reduced time exposures to SIN-1 (4 h: *p* < 0.001). Significant protective actions were also observed for 4HPP (10 μM; *p* < 0.01) (Figure 1B); 3HPP (10 μM, *p* < 0.05) (Figure 1C) and 3HPA (0.1–10 μM, *p* < 0.05) (Figure 1D), when SIN-1-induced toxicity was less prolonged (4–6 h). A similar trend was observed for both salicylic acid (Figure 1E) and its glucuronide (Figure 1F), which were shown to be protective against SIN-1 after 4 h exposure, but innefective for longer periods (6–15 h). All concentrations of glucuronide of salicylic acid tested were effective at maintaining cell viability after 4 h of SIN-1 exposure (0.1 μM, *p* < 0.001; 1–10 μM, *p* < 0.05) (Figure 1F), while for salicylic acid, only 1 μM concentration resulted in a significant increase in cell survival (*p* < 0.05) (Figure 1E).

Aroma compounds 1,8-cineole and linalool (Figures 1G,H) showed a neuroprotective effect at specific concentrations and specific times of exposure. 1,8-Cineole was able to increase cell viability after 9 h SIN-1 incubation (*p* < 0.01) (Figure 1G), while linalool was only effective after shorter periods of SIN-1 exposure (4 h; *p* < 0.05) (Figure 1H).

### Effect of Phenolic Acids and Aroma Compounds on SIN-1-Induced p38 Phosphorylation

**Figure 2:**
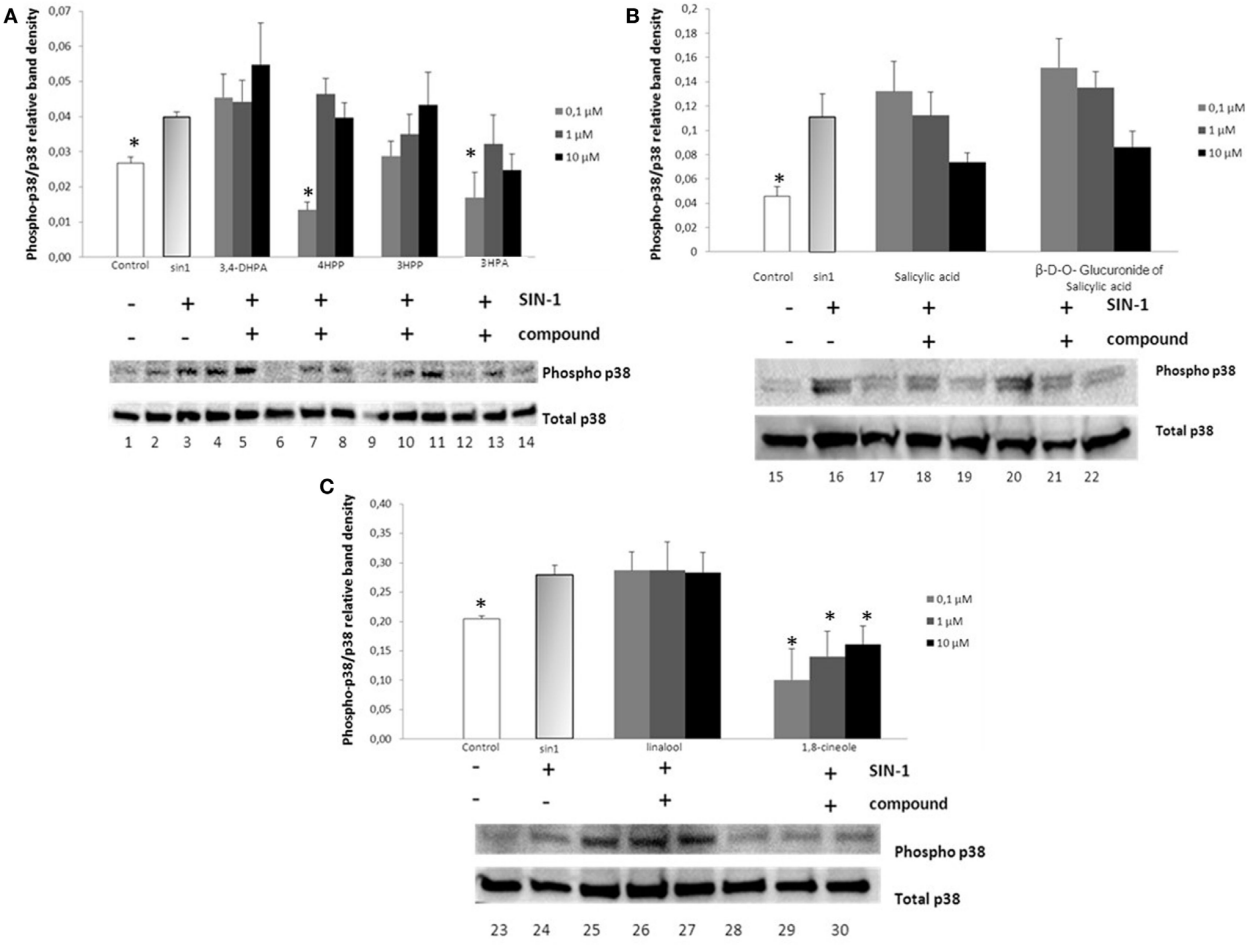
**Inhibition of p38 phosphorylation in SIN-1 activated SH-SY5Y cells**. Cells were treated with SIN-1 (500 μM) for 1 h in all the cases except for salicylic acid and its glucuronidie that was exposed for 2 h, after being pretreated with wine compounds (0.1–10 μM) for 18 h. Crude homogenates (20 μg) were immunoblotted with antibodies that detect endogenous levels of p38 only when phosphorylated. Data obtained from immunoblot experiments with p38 were analyzed using Image Quant LAS 4000 mini software, and each column represents the mean ± SEM of three independent experiments. **(A)** Phosphorylation status of p38 after incubation with phenolic acids [DHPA, 4HPP, 3HPP, and 3-hydroxyphenylacetic (3HPA)]. 1: control; 2: SIN-1; 3–5: DHPA (0.1, 1, and 10 μM, respectively); 6–8: 4HPP (0.1, 1, and 10 μM, respectively); 9–11: 3HPP (0.1, 1, and 10 μM, respectively); 12–14: 3HPA (0.1, 1, and 10 μM, respectively). * Indicates values significant different (*p* < 0.05) from SIN-1 group as analyzed by one-way analysis of variance (ANOVA) followed by Dunnett comparison test. **(B)** Phosphorylation status of p38 after incubation with salicylic acid and its glucuronide. 15: control; 16: SIN-1; 17–19: salicylic acid (0.1, 1, and 10 μM, respectively); 20–22: β-d-O-glucuronide (0.1, 1, and 10 μM, respectively). * Indicates values significant different (*p* < 0.06) from SIN-1 group as analyzed by one-way ANOVA followed by Dunnett comparison test. **(C)** Phosphorylation status of p38 after incubation with aroma compounds. 23: control; 24: SIN-1; 25–27 linalool (0.1, 1, and 10 μM, respectively); 28–30 1,8-cineole (0.1, 1, and 10 μM, respectively). *p < 0.05 indicates values significant different (except *p* < 0.06 in the case of control) from SIN-1 group as analyzed by one-way ANOVA followed by Dunnett comparison test.

Exposure of SH-SY5Y cells to SIN-1 (1 h in all the cases, except for salicylic acid and its glucuronide that was exposed for 2 h; 500 μM) resulted in a significant increase in the phosphorylation of p38 when compared to the control (*p* < 0.05) (Figure 2). Treatment of the cells with wine-derived compounds (0.1–10 μM for 18 h) prior to exposure to SIN-1 only resulted in a significant inhibition of p38 phosphorylation status for 4HPP (*p* < 0.01) and 3HPA (*p* < 0.05) at the lower concentration (0.1 μM) (Figures 2A,B). The aroma compound 1,8-cineole also exerted a significant inhibition of p38 at all concentrations tested (0.1–10 μM; *p* < 0.05) (Figure 2C).

### Effect of Phenolic Acids and Aroma Compounds on SIN-1-Induced ERK1/2 Phosphorylation

**Figure 3:**
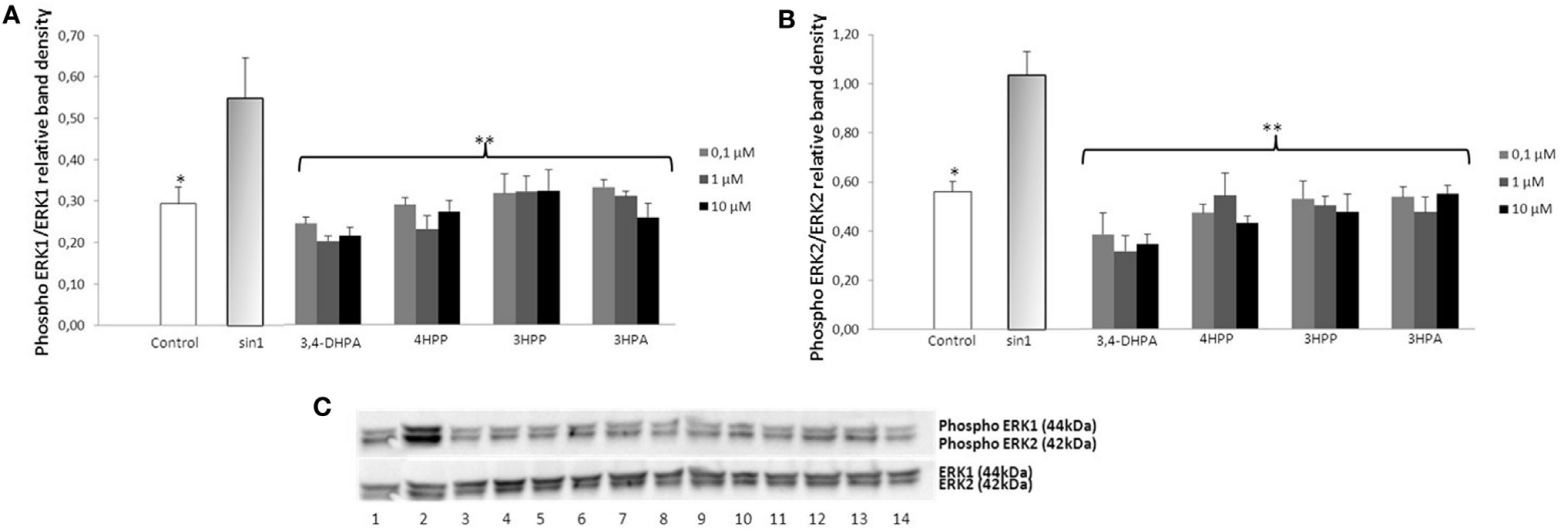
**Inhibition of ERK1/2 phosphorylation in SIN-1 activated SH-SY5Y cells**. **(A)** Data obtained from immunoblot experiments with ERK1 were analyzed using Image Quant LAS 4000 mini software and each column represents the mean ± SEM of three independent experiments. *p < 0.05, **p < 0.01 indicate values significant different from SIN-1 group as analyzed by one-way analysis of variance (ANOVA) followed by Dunnett comparison test. **(B)** Data obtained from immunoblot experiments with ERK2 were analyzed using Image Quant™ Software, and each column represents the mean ± SEM of three independent experiments. *p < 0.05, **p < 0.01 indicate values significant different from SIN-1 group as analyzed by one-way ANOVA followed by Dunnett comparison test. **(C)** Western blot of the phenolic acids (DHPA, 4HPP, 3HPP, and 3-hydroxyphenylacetic (3HPA)) inhibition of ERK1/2 phosphorylation in SIN-1-activated neuronal cells. Cells were treated with SIN-1 (500 μM) for 1 h after being pretreated with phenolic acids (0.1–10 μM) for 24 h. Crude homogenates (20 μg) were immunoblotted with antibodies that detect endogenous levels of ERK 1/2 only when phosphorylated. 1: control; 2: SIN-1; 3–5: DHPA (0.1, 1, and 10 μM, respectively); 6–8: 4HPP (0.1, 1, and 10 μM, respectively); 9–11: 3HPP (0.1, 1, and 10 μM, respectively); 12–14: 3HPA (0.1, 1, and 10 μM, respectively).

**Figure 4:**
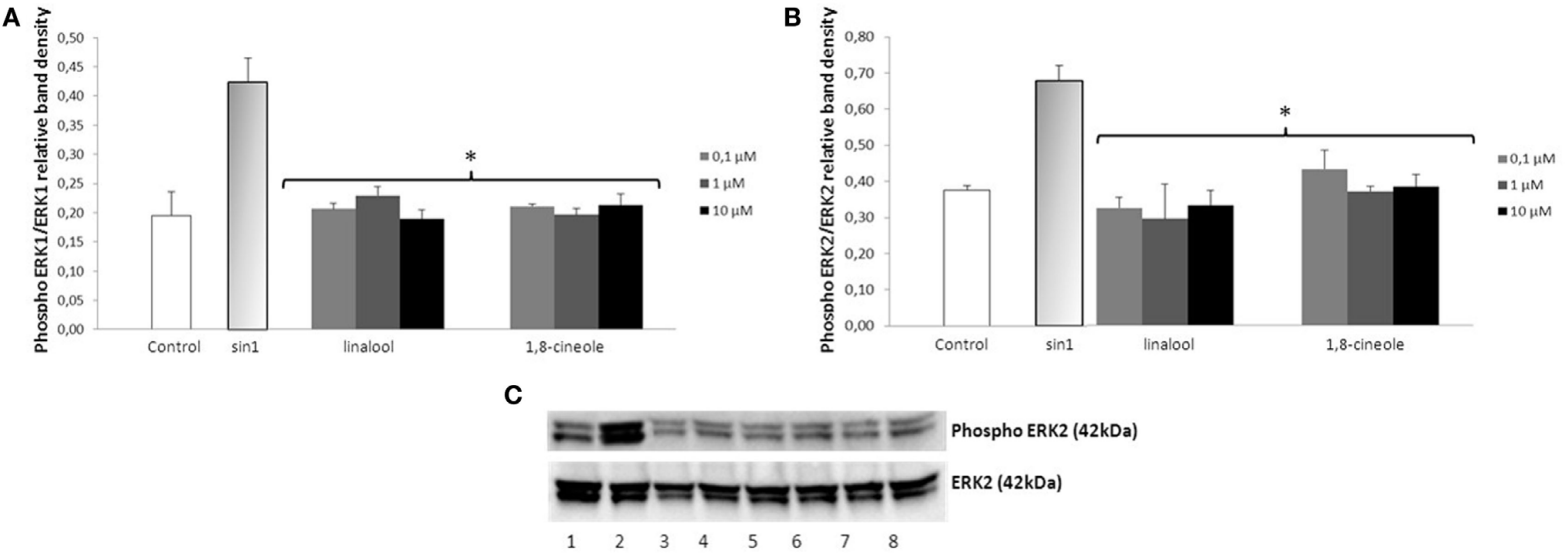
**Inhibition of ERK1/2 phosphorylation in SIN-1 activated SH-SY5Y cells**. **(A)** Data obtained from immunoblot experiments with ERK1 were analyzed using Image Quant LAS 4000 mini software and each column represents the mean ± SEM of three independent experiments. *p < 0.05 indicates values significant different from SIN-1 group as analyzed by one-way analysis of variance (ANOVA) followed by Dunnett comparison test. **(B)** Data obtained from immunoblot experiments with ERK2 were analyzed using Image Quant™ Software, and each column represents the mean ± SEM of three independent experiments. *p < 0.05 indicates values significant different from SIN-1 group as analyzed by one-way ANOVA followed by Dunnett comparison test. **(C)** Western blot of the aroma compounds (linalool and 1,8-cineole) inhibition of ERK1/2 phosphorylation in SIN-1-activated neuronal cells. Cells were treated with SIN-1 (500 μM) for 1 h after being pretreated with aroma compounds (0.1–10 μM) for 24 h. Crude homogenates (20 μg) were immunoblotted with antibodies that detect endogenous levels of ERK 1/2 only when phosphorylated. 1: control; 2: SIN-1; 3–5: linalool (0.1, 1, and 10 μM, respectively); 6–8: 1,8-cineole (0.1, 1, and 10 μM, respectively).

Exposure of neuroblastoma cells to SIN-1 (1 h; 500 μM) leads to a significant increase of ERK1/2 phosphorylation when compared to the control (*p* < 0.05) (Figures 3 and 4). Pretreatment with wine-derived phenolic acids 3,4-DHPA, 4HPP, 3HPP, and 3HPA resulted in a significant decrease in ERK1/2 phosphorylation at all concentrations tested (*p* < 0.01), reaching similar levels as control (Figure 3). Similarly, the aroma compounds, linalool and 1,8-cineole exerted a significant reduction in ERK1/2 activation (at all concentrations tested) (*p* < 0.05) in relation to the SIN-1 group (Figure 4). Regarding salicylic acid and its glucuronide, no modulatory effect over ERK1/2 phosphorylation was observed (data not shown). We observed no modulation of JNK and STAT-1 by SIN-1 or any of the wine-derived compounds tested (data not shown).

### Effect of Phenolic Acids and Aroma Compounds in Caspase-3 Activation Following SIN-1-Induced Neuronal Damage

**Figure 5:**
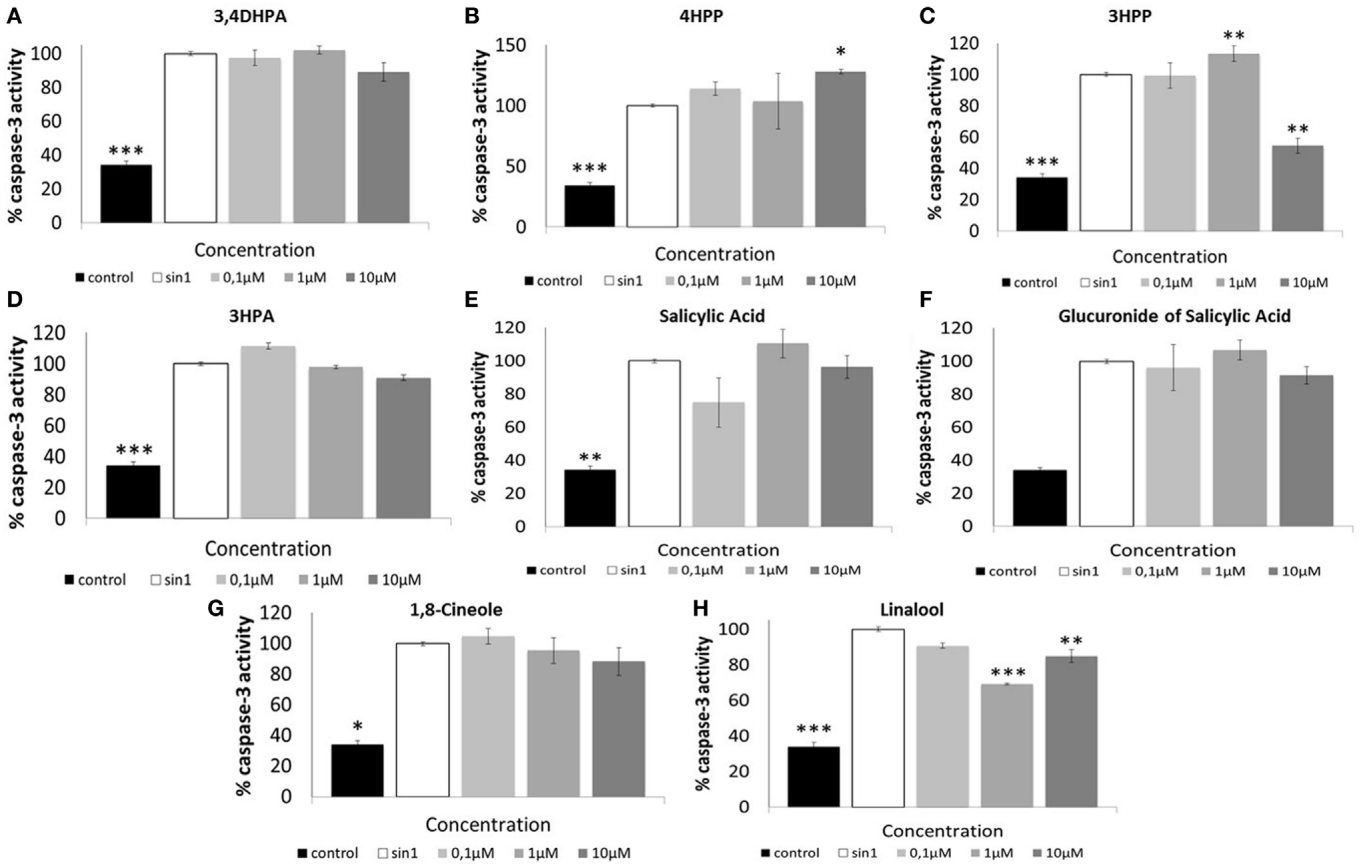
**Relative activity of Caspase-3 protein (%) in SH-SY5Y cells after SIN-1-induced damage**. Cells were pretreated with phenolic **(A–F)** and aroma compounds **(G,H)** or media (control) for 18 h before SIN-1 incubation. After 6 h exposure, cells were washed, scraped, and lysed. Lysates were analyzed by Caspase-3 colorimetric assay. Data are indicated as% of caspase-3 activity, considering SIN-1 group the maximum activity for this protein (100%). Results are expressed as means of three independent experiments ± SEM. *p < 0.05, **p < 0.01, ***p < 0.001 indicate values significant different from SIN-1 group as analyzed by one-way ANOVA followed by Dunnett comparison test.

Treatment of SH-SY5Y cells with SIN-1 (6 h; 1 mM) resulted in a significant increase of caspase-3 activation in relation to control (*p* < 0.001) (Figure 5). When administered at 10 μM, phenolic acid 3HPP reduced significantly caspase-3 activation (*p* < 0.01) in relation to SIN-1 control (Figure 5C). The aroma compound linalool (1, 10 μM) also showed a significant reduction (*p* < 0.01 and *p* < 0.001, respectively) in the activation of caspase-3 in relation to SIN-1-treated cells (Figure 5H).

### Effect of MAPK Signaling Inhibitors in Cell Viability and Caspase-3 Activation after SIN-1-Induced Neuronal Damage

**Figure 6:**
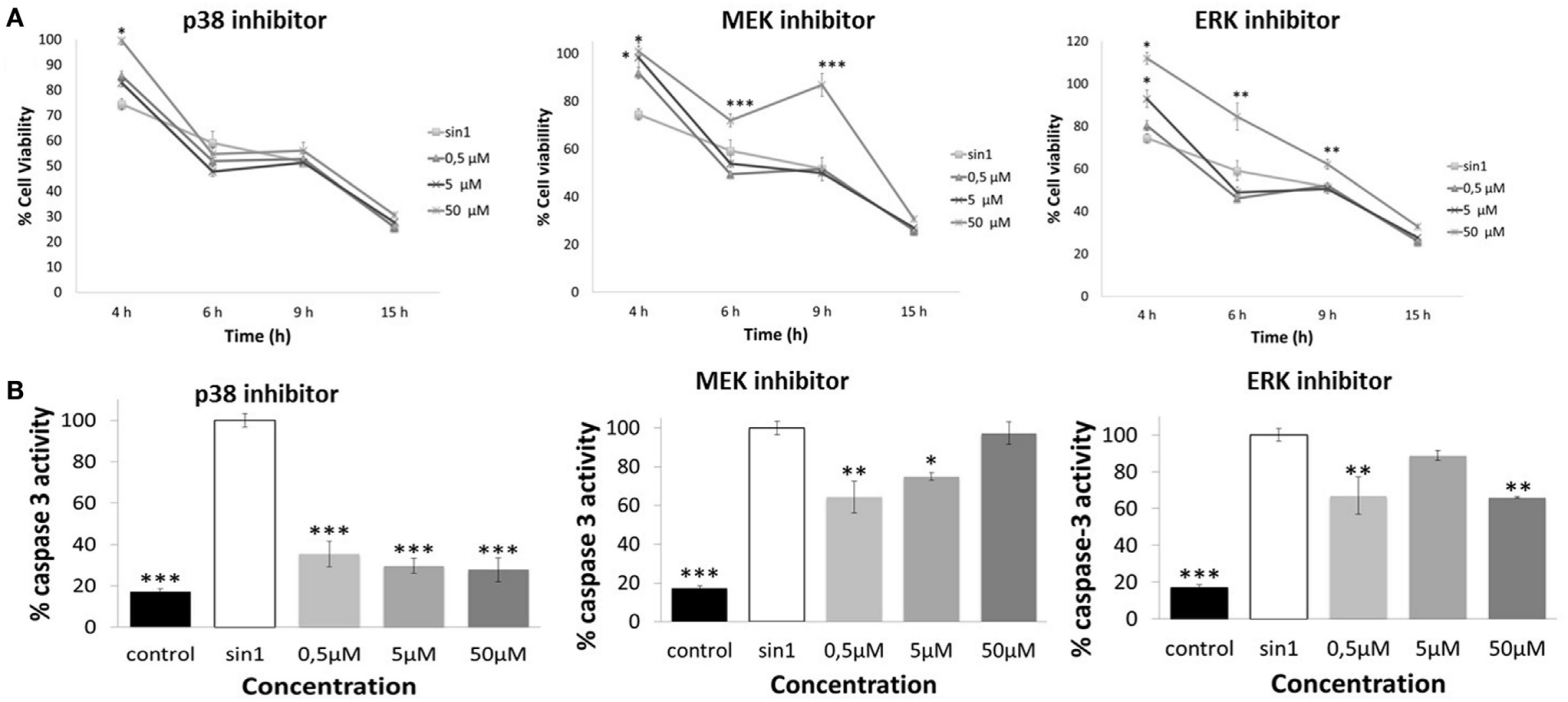
**(A)** Neuroprotective effects of p38 inhibitor (SB203580), MEK inhibitor (PD98059), and ERK inhibitor (FR180204) against SIN-1-induced damage in SH-SY5Y cells. Following pretreatment with inhibitors for 1 h or media (control), cells were exposed to SIN-1 (1 mM) for 0–15 h, before assessment of viability by MTT assay. Results are expressed as means of three independent experiments ± SEM. *p < 0.05, **p < 0.01, ***p < 0.001 indicate values significantly different from SIN-1 group (zero) as analyzed by one-way analysis of variance (ANOVA) followed by Dunnett comparison test. **(B)** Relative activity of Caspase-3 protein (%) in SH-SY5Y cells after SIN-1-induced damage. Cells were pretreated with inhibitors for 1 h before SIN-1 incubation. After 6 h exposure, cells were washed, scraped, and lysed. Lysates were analyzed by Caspase-3 colorimetric assay. Data are indicated as% of caspase-3 activity, considering SIN-1 group the maximum activity for this protein (100%). Results are expressed as means of three independent experiments ± SEM. *p < 0.05, **p < 0.01, ***p < 0.001 indicate values significant different from SIN-1 group as analyzed by one-way ANOVA followed by Dunnett comparison test.

Inhibition of ERK1/2 (FR180204) and upstream MEK1 (PD98059) significantly increased cell viability (*p* < 0.05), with the highest concentration tested being the most effective (50 μM) against (4–15 h) SIN-1-induced toxicity (Figure 6A). Inhibition of p38 (SB203580) resulted in an increased of cell viability (at 4 h SIN-1 incubation) only when administered at the highest concentration (50 μM) (*p* < 0.05) (Figure 6A).

Furthermore, inhibition of p38, MEK, and ERK1/2 resulted in an overall significant reduction of caspase-3 activation, which confirms that SIN-1-induced MAPK signaling is linked to the activation of caspase-3 protein (Figure 6B). Specifically, p38 inhibition consistently decreased caspase-3 protein activation, with all the concentrations tested being equally effective (*p* < 0.001). For MEK and ERK1/2 not all concentrations resulted in a significant reduction in caspase-3 activity. In particular, MEK inhibitor concentrations were effective in reducing caspase-3 activity only when administered at 0.5 and 5 μM (*p* < 0.05), while ERK1/2 inhibitor doses were effective at 0.5 and 50 μM (*p* < 0.01).

## Discussion

Specific wine-derived phenolic compounds were selected for the present study given their bioavailability in urine and feces after moderate and regular wine consumption in humans (31, 32). In support of our model, Gasperotti and colleagues (16) recently reported that phenolic acids 3HPA, 3,4-DHPA, and HPP were present in the brain of rats following intake of 23 polyphenol microbial metabolites. Similarly, Wang et al. (17) observed that 3-hidroxybenzoic acid and 3HPP were also accumulated in rodent brains at μM concentrations after orally administration of grape seed polyphenols extract. Altogether, this strongly suggests that microbial-derived phenolic acids can cross the BBB *in vivo*, and therefore, they have the potential to act centrally. The stress induced by SIN-1 led to a significant and time-dependent cell death in SH-SY5Y cell line. In a similar manner, SIN-1 treatment resulted in a significant phosphorylation of ERK1/2 and p38 MAPK proteins, as well as downstream caspase-3 protein activation. In agreement with our results, other assays in the same cellular model have demonstrated that peroxynitrite is able to induce neuronal damage *via* ERK1/2 and p38 modulation, as well as to induce caspase-3 protein activation (29, 30, 33, 34), which validates our model.

In this study, 3,4-DHPA showed a strong protective effect against SIN-1 induced-cell death, especially at 10 μM. This is in agreement with previous studies where 3,4-DHPA, at similar doses (20 μM), completely blocked the effect of peroxynitrite on tyrosine hydroxylase, an enzyme involved in Parkinson’s disease pathology (35). Furthermore, recent evidences showed a preventive action of this phenolic metabolite in dysfunctional pancreatic-β-cells (36), even at not physiologically relevant concentrations (up to 250 μM), as well as on mice liver after intragastrically administration of 3,4-DHPA (10, 20, or 50 mg/kg) for 3 days (37). Additionally, in the meanline of the redaction process of this paper, new evidence was reported showing the ability of a panel of phenolic metabolites, noting 3,4-DHPA among them, to prevent neuronal death after oxidative (H_2_O_2_) induced injury in the SH-SY5Y cellular model at a similar range of concentrations (1–10 μM) (38). β-d-*O*-Glucuronide of salicylic acid also showed a significant neuroprotective effect in the present study, unlike what was observed for its aglycone as González-Sarrias et al. (38) also reported. In disagreement with the weaker ability to access cells of glucuronide forms (39), some studies proposed the possibility of β-glucuronidases cleaving the glucuronide form and releasing the free aglycone (40). Further significant reductions of cell death were also seen for 4HPP, 3HPP, 3HPA, linalool, and 1,8-cineole, particularly at shorter incubation times. In similar conditions, previous research showed a neuroprotective effect of champagne wine polyphenols, including gallic acid, caffeic acid, and tyrosol (0.1 and 10 μM) on primary cortical neurons (41) after SIN-1-induced damage and after *S*-cysteinyl-dopamine toxin exposition (42), as well as (−)-epicatechin oligomers have been described to neutralize peroxynitrite damage (0–20 μM) (43).

When pro-inflammatory signals are triggered in the central nervous system following nitrosative stress, MAPK kinases, such as ERK1/2, JNK, and p38 (24), become phosphorylated, leading to the activation of transcription factors, such as STAT-1 (1), and favoring the activation of proteins related to cellular damage, inflammation, and apoptosis, such as caspase-3. p38 protein has been related to neuronal stress and the control of cell death and survival (44), while the role of ERK1/2 on this balance is controversial. This protein has been usually linked to a pro-survival function (45); however, several studies demonstrated a role of ERK1/2 on oxidative stress-induced apoptosis (33, 46–48). This is in agreement with our results, where an increase of pERK1/2 was observed after SIN-1 treatment. Additionally, the use of ERK1/2 specific inhibitor resulted in an increase of cell viability after SIN-1 induced-stress. All the phenylacetic and phenylpropionic acids (3,4-DHPA, 4HPP, 3HPP, and 3HPA) and aroma compounds (linalool and 1,8-cineole) tested showed a modulatory effect on SIN-1 induced-phosphorylation of ERK1/2. On the other hand, no modulation of ERK1/2 was observed for salicylic acid and its glucuronide, which might indicate that they act through alternative pathways, such as NF-κB, described to be modulated by a salicylic acid analog (49). In addition, 4HPP and 3HPA (0.1 μM), as well as 1,8-cineole (0.1–10 μM) also significantly modulated p38 phosphorylation. Several lines of research have demonstrated a modulation of MAPK by a wide range of polyphenols (24) especially, flavonoids (1, 24, 50). For instance, naringenin was able to modulate p38 phosphorylation (0.01–0.3 μM) in a neuroinflammation primary mixed glial cells model (25). Similarly, (−) epicatechin is also able to interact with JNK and ERK1/2 at physiologically relevant concentrations to protect neurons against oxidative stress-induced death (51). However, these studies have mainly focused on the effects of aglycones (24), not in physiologically relevant microbial metabolites that are found in the circulation following polyphenols-rich food intake (38, 52). As exception, the protocatechuic acid (3,4-dihydroxybenzoic acid), was shown to modulate inflammation *via* modulation of BDNF, resulting in amelioration of cognitive deficits, as well as to attenuate amyloid deposits in aged AβPP/PS1 double transgenic mice (100 mg/kg/day during 4 weeks) (53). Regarding aroma compounds, both 1,8-cineole and linalool, have been previously described to modulate *in vitro* stress-induced signaling pathways, such as NF-kB (21, 22). Concurrently, and in agreement with our results, 1,8-cineole was described to interact with p38 MAPK protein in an asthma model of bronchial epithelial cells (54). These observations were *in vivo* corroborated, with the exposure to 1,8-cineole resulting in better cognitive performance in a pilot clinical human study (*n* = 20) (20). Additionally, oral administration of linalool (25 mg/kg for 3 months) to Alzheimer’s triple transgenic mice showed a beneficial effect on behavioral impairment, as well as a reduction in pro-inflammatory markers (p38 MAPK, NOS2, COX2, and IL-1β) (55).

Caspase-3 protein promotes stress-mediated apoptosis by endonucleases activation (27). Flavonoids, such as (−)-epicatechin (and some derivatives as 3′-*O*-methyl-epicatechin and (−)-epicatechin-glucuronide) and kaempferol were reported to reduce caspase-3 levels on cultured striatal neurons and fibroblasts (30 μM) (39, 56). We further observed significant reductions on downstream active caspase-3 levels after pretreatment with 3HPP and linalool, despite p38 activation levels were not affected. The observed fact of the reduction of p38 and ERK1/2 activation not being accompanied by an amelioration of caspase-3 activity has been also previously perceived in other *in vitro* models of induced stress (33). This could suggest that the most part of these compounds are not able of affecting this point but also that caspase-3 can become activated in several scenarios, such as mitochondrial damage and cytochrome *c* release (57).

Finally, we observed that pretreatment of MEK-, ERK1/2-, and p38-specific inhibitors with a phenolic-like structure resulted in an increase on cell survival and a reduction on caspase-3 levels. These were similar to the effects we observed for phenolic acids and the aroma compounds tested, suggesting that wine compounds could exert a neuroprotective effects by inhibiting ERK1/2 and p38, as well as by modulating downstream caspase-3 protein. The application of specific MAPK inhibitors has been widely used with the aim of establishing mechanism of actions of polyphenols, based on its structural homology (24, 56).

**Figure 7:**
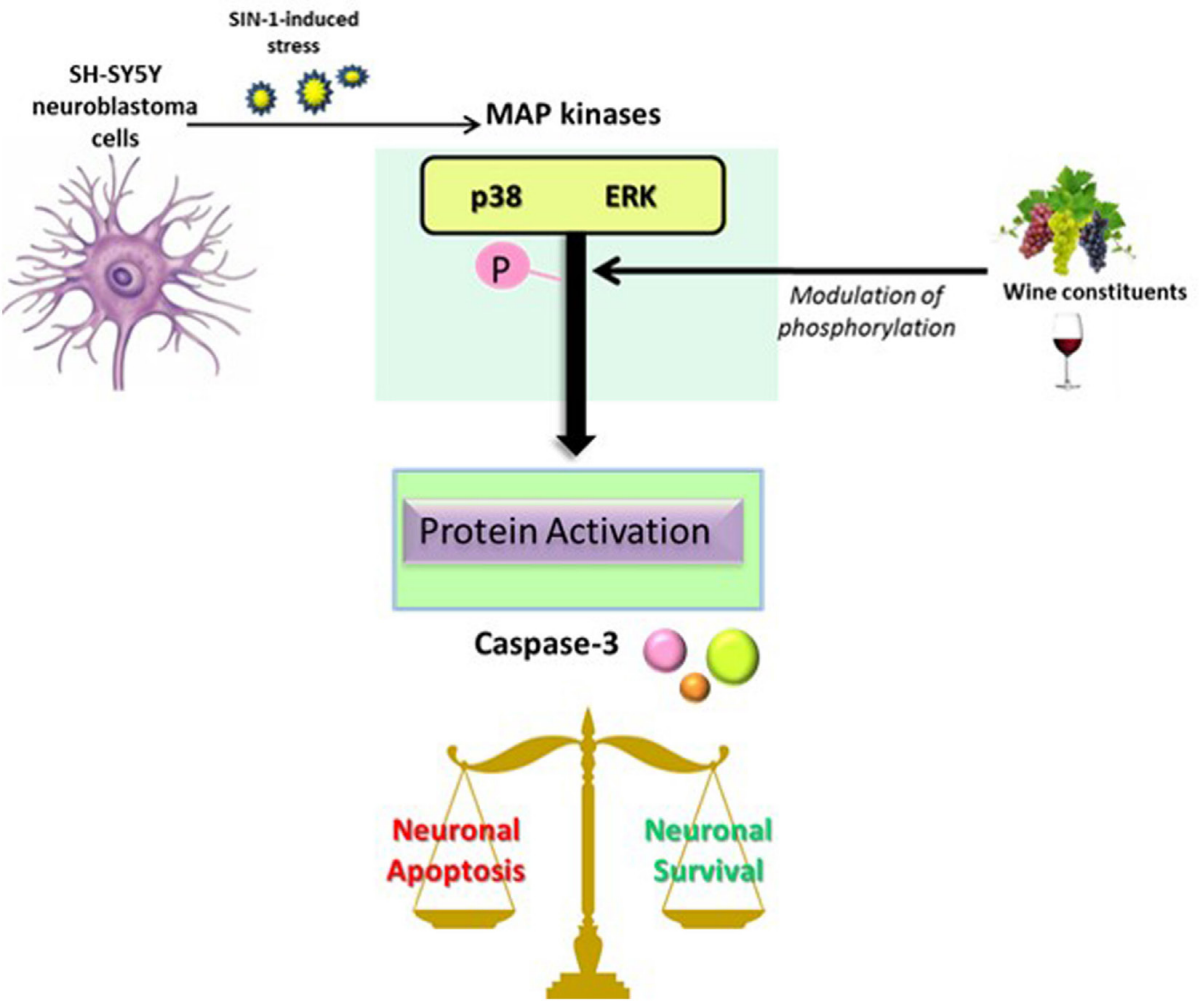
**Proposed scheme of the mitogen-activated protein kinase (MAPK) signaling cascade after a stress-induced phosphorylation in neurons and its implication in the balance of cell apoptosis and survival signals**. Phosphorylation of MAPK (p38 or ERK) leads to the activation of several proteins, including caspase-3, and therefore resulting in a modification of the balance between apoptotic and pro-survival signals in neurons. The activation of MAPK could be modified by the action of wine constituents, including phenolic acids and aroma compounds.

In summary, in the present study, we demonstrate that specific wine-derived phenolic acids and aroma compounds, in particular 3,4-DHPA, are protective against neuronal death induced by SIN-1 in a dopaminergic cell line. Such protective actions are likely modulated *via* inhibition of ERK1/2 (for aroma compounds and all phenolic acids, except for salicylic acid and its glucuronide, which suggest other mechanisms of action for these compounds), and *via* modulation of p38 MAPK kinases (1,8-cineole, 4HPP, and 3HPA), as well as through downstream pro-apoptotic caspase 3 activation (3HPP and linalool) (Figure 7). In support of this, we further show that pharmacological inhibition of MEK/ERK and p38 in this model, results in similar protective effects on cell viability and also in a reduction of caspase 3 activation, strongly suggesting that protective actions of phenolic acids might be linked to these pathways.

## Author Contributions

MM-A and BB conceived and designed the study, analyzed the data, and wrote the paper. AE-F and DC performed the experiments, and AE-F participated in the redaction of the manuscript. JS, DC, and CR participated in the design and critical revision. All authors read and approved the final manuscript.

## Conflict of Interest Statement

The authors declare that the research was conducted in the absence of any commercial or financial relationships that could be construed as a potential conflict of interest.

## Abbreviations

3,4-DHPA, 3,4-dihydroxyphenylacetic acid; 4HPP, 3-(4-hydroxyphenyl) propionic acid; 3HPP, 3-(3-hydroxyphenyl) propionic acid; 3HPA, 3-hydroxyphenylacetic acid; SIN-1, 3-morpholinosydnonimine; MAPK, mitogen-activated protein kinases.
